# MRI Changed the diagnosis of prostate cancer

**DOI:** 10.1590/S1677-5538.IBJU.2023.0354.1

**Published:** 2024-07-25

**Authors:** Luciano A. Favorito

**Affiliations:** 1 Universidade do Estado do Rio de Janeiro Unidade de Pesquisa Urogenital Rio de Janeiro RJ Brasil Unidade de Pesquisa Urogenital - Universidade do Estado do Rio de Janeiro - Uerj, Rio de Janeiro, RJ, Brasil; 2 Hospital Federal da Lagoa Rio de Janeiro RJ Brasil Serviço de Urologia, Hospital Federal da Lagoa, Rio de Janeiro, RJ, Brasil

## COMMENT

In cases of prostate cancer investigation MRI can be used during a prostate biopsy to help guide the needles into the prostate. This paper of the group of Dr. Abreu and Gill ( 1 ) shows the importance of MRI in the prostate cancer diagnosis. In the last years several papers studied the prostate biopsy showing the superiority of the perineal MRI/TRUS Fusion-Guided Prostate Biopsy ( 2 – 4 ). The group of the University of Southern California compared transperineal (TP) vs transrectal (TR) magnetic resonance imaging (MRI) and transrectal ultrasound (TRUS) fusion-guided prostate biopsy (PBx) in a large, ethnically diverse and multiracial cohort with 1491 patients, with 480 undergoing TP and 1011 TR PBx and concluded that in a large and diverse cohort, black race, but not the biopsy approach, was an independent predictor for clinical significant prostate cancer (CSPCa) detection. TP and TR PBx yielded similar CSPCa detection rates, however the TP PBx was histologically more informative.

## References

[1] Ramacciotti LS, Strauss D, Cei F, Kaneko M, Mokhtar D, Cai J, Jadvar D, Cacciamani GE, Aron M, Halteh PB, Duddalwar V, Gill I, Abreu AL (2024). Transperineal versus Transrectal MRI/TRUS fusion-guided prostate biopsy in a large, ethnically diverse, and multiracial cohort. Int Braz J Urol.

[2] Lv Z, Wang J, Wang M, Hou H, Song L, Li H (2023). Is it necessary for all patients with suspicious lesions undergo systematic biopsy in the era of MRI-TRUS fusion targeted biopsy?. Int Braz J Urol.

[3] Gilberto GM, Arcuri MF, Falsarella PM, Mariotti GC, Lemos PLA, Garcia RG (2023). Complication rates of transrectal and transperineal prostate fusion biopsies - is there a learning curve even in high volume interventional center?. Int Braz J Urol.

[4] Andrade GM, Sesconetto L, da Silva RBR, Dos Santos GGR, Kayano PP, Baccaglini W (2023). Impact of COVID-19 pandemic on prostate cancer outcomes at an uro-oncology referral center. Int Braz J Urol.

